# Assessment of the physicochemical characteristics, chemical and microbiological safety of two types of *kilichi*, a grilled meat produced in Niger

**DOI:** 10.1002/fsn3.1190

**Published:** 2019-09-03

**Authors:** Ramatou Boubacar Seydou, AbdoulKader Harouna, Yénoukounmè Euloge Kpoclou, Caroline Douny, François Brose, Marichatou Hamani, Jacques Mahillon, Victor Bienvenu Anihouvi, Marie‐Louise Scippo, Djidjoho Joseph Hounhouigan

**Affiliations:** ^1^ Laboratory of Food Sciences Faculty of Agronomic Sciences School of Nutrition and Food Science and Technology University of Abomey‐Calavi Cotonou Benin; ^2^ Institut National de Recherche Agronomique Niamey Niger; ^3^ Laboratory of Food Analysis, Fundamental and Applied Research for Animals & Health Department of Food Sciences Faculty of Veterinary Medicine University of Liège Liège Belgium; ^4^ Département de Productions Animales Faculté d'Agronomie Université Abdou Moumouni Niamey Niger; ^5^ Laboratory of Food and Environmental Microbiology Faculty of Bioscience Engineering, Earth and Life Institute Catholic University of Louvain Louvain‐la‐Neuve Belgium

**Keywords:** microbiological contamination, polycyclic aromatic hydrocarbons, quality, risk assessment

## Abstract

Production of *kilichi*, a grilled meat of West Africa, is a common method of meat preservation in Niger. Thirty samples of condiments‐coated *kilichi* and uncoated *kilichi* collected in Niger, were analyzed for microbiological contamination, as well as NaCl, protein and lipid contents, using standard methods. Contamination with Polycyclic Aromatic Hydrocarbons (PAHs) was also assessed using a HPLC–FLD technique. Highly significant differences (*p* < .001) were observed between coated *kilichi* and uncoated *kilichi,* for NaCl content (2.56% and 1.40%), for proteins (51% and 72%) and lipids (18% and 13%), respectively. Water activity was low in both *kilichi*, showing a potential microbial stability. Among the 15 European Union (EU) priority PAHs, 12 were detected in the samples. About 56.3% of coated *kilichi* samples exceeded the EU maximal limit for BaP, and 75% exceeded the EU maximal limit for the sum of 4 PAHs (PAH4). For uncoated *kilichi*, 28.6% of samples did not meet the standards for BaP and PAH4. About 6% of coated *kilichi* samples were not compliant with standards related to *Staphylococcus aureus*, *Bacillus cereus, Clostridium perfringens*, and 31%, 50% for yeasts and fungi, respectively. *Escherichia coli* and Enterobacteria were below the detection limit in both *kilichi*, but *Salmonella* and *Bacillus cereus* were detected only in one coated *kilichi*. The noncompliant samples of uncoated *kilichi* were in the proportions varying between 7% –86% for *S. aureus, C. perfringens,* yeasts, and fungi. This study showed potential risks associated with the consumption of traditionally produced *kilichi* in Niger due to both PAHs and pathogen bacteria contamination.

## 1 INTRODUCTION

Breeding plays a central role in the countries' economy of the Sahelian zone of West Africa, where it constitutes an important source of income for millions of rural or periurban populations whose mean of existence is linked to pluvial agriculture (Kamuanga et al., 2008). Breeding has favored the development of a dynamic artisanal food processing sector producing and selling a number of meat products, and providing substantial revenues for producers and animal proteins for populations.

Among these products, *kilichi*, a dried meat, coated with condiments or uncoated, then grilled, is well appreciated by consumers in local markets in Niger (Beidari & Mahamadou, 2014). *Kilichi* is a ready‐to‐eat meat product traditionally manufactured with bovine, camel, ovine, or goat fresh meat. Coated *kilichi* is produced by trimming meat, cutting into pieces of parallelepiped shape before slicing into flat thin sheets. The sheets are spread on millet or sorghum panicle mats for a first sun drying followed by marinating in a sauce made of complex blend of spices, before a second sun drying and briefly grilling at wood fire. For uncoated *kilichi*, the sun dried meat is slightly seasoned and grilled. *Kilichi* is generally packaged, only just before selling to consumers (Boubacar et al., 2019). The conditions of processing and distribution practices do not guaranty the safety of this product. Analyses conducted on this product in Tchad and Cameroun showed high levels of contamination by pathogenic bacteria, in particular *Salmonella*, presenting a risk of acute intoxication for consumer (Kimassoum et al., 2017; Mbawala, Daoudou, & Ngassoum, 2010). The problem of pathogenic bacteria seems to be the most worrying, but grilling step of the product shows risk of contamination by Polycyclic Aromatic Hydrocarbons (PAHs). In fact, PAHs are toxic chemical contaminants generated during the combustion of organic material. Food products mostly get chemical contamination through thermal processing such as smoking, roasting, grilling, frying, and drying where they are in direct contact with the combustion source (Farhadian, Jinap, Abas, & Sakar, 2010; Rose et al., 2015; Roseiro, Gomes, Patarata, & Santos, 2012). At high concentration, PAHs could be carcinogenic and genotoxic. Some respiratory, cardiologic, immunologic, neurologic, reproductive, and genotoxic imperfections in human and animals are linked to the harmful effect of PAHs (EFSA, 2008; Olabemiwo & Ogunsola, 2014). The parameters like temperature, time, distance, biomass, relative humidity, and characteristics of the products have effect on the absorption and penetration of smoke components in product, and therefore, on its quality and stability (Akpambang et al., 2009; Santos, Gomes, & Roseiro, 2011).

The Scientific Committee on Food (SCF) of the European Commission (2002) identified 15 Polycyclic Aromatic Hydrocarbons (15 EU PAHs) as carcinogenic and genotoxic, which are Benzo[a]anthracene (BaA), Benzo[b]fluoranthene (BbF), Benzo[j]fluoranthene (BjF), Benzo[k]fluoranthene (BkF), Benzo[a]pyrene (BaP), Benzo[g,h,i]perylene (BgP), Chrysene (CHR), Cyclopenta[c,d]pyrene (CCP), Dibenzo[a,h]anthracene (DhA), Dibenzo[a,e]pyrene (DeP), Dibenzo[a,h]pyrene (DhP), Dibenzo[a,i]pyrene (DiP), Dibenzo[a,l]pyrene (DIP), Indeno[1,2,3‐cd]pyrene (IcP), and 5‐methylchrysene (5MC). On the recommendation of the Joint FAO/WHO Expert Committee on Food Additives (JECFA, 2005) a 16th compound, Benzo[c]fluorene (BcF), has been added to the list. Little has been done on the characterization of *kilichi* in Niger, particularly on physicochemical, nutritional, and microbiological aspects, and to our knowledge, no study on the contamination by PAHs. Therefore, the aim of this work was to assess the nutritional, chemical, and microbiological characteristics of *kilichi* produced in Niger, and to estimate the risks associated with the consumption of PAHs contaminated *kilichi* for the purpose of improving its manufacturing process and quality.

## 2 MATERIALS AND METHODS

### 2.1 Sampling

The sampling was carried out in the regions of Agadez, Maradi, Niamey, Tahoua, & Zinder. About 30 samples of uncoated and coated *kilichi* were randomly bought at 17 manufacturing sites and markets of the regions as distributed in Table 1. The samples were composed of 14 uncoated samples (11 of beef and 3 of camel) and 16 coated samples (13 of beef and 3 of camel). Each collected sample was packaged in a sterile stomacher bag and stored in refrigerator for microbiological analysis and in freezer at −20°C for physicochemical analyses and determination of PAHs.

**Table 1:** Distribution of *kilichi* samples collected in different regions of Niger for analyses

Sampling areas (Region)	*kilichi* types		Total
	Uncoated *kilichi*	Coated *kilichi*	
Agadez	3	3	6
Maradi	3	3	6
Niamey	2	4	6
Tahoua	3	3	6
Zinder	3	3	6
Total	14	16	30

### 2.2 Physicochemical analyses

The physicochemical parameters determined were: pH, moisture, water activity, protein, fat, ash, and NaCl contents. The pH was measured as described by Mgbemere, Akpapunam, and Igene (2011). The moisture of samples was determined using the ISO 1442/1997 standard. The water activity (a_w_) was measured according to the method described by Anihouvi, Ayerno, Hounhouigan, and Sakyi‐Dawson, (2006) using a hygrometer (HygroLab, model Rotronic). The nitrogen content of samples was determined according to ISO 937/1978 standard, and the protein content was calculated by multiplying the nitrogen content by 6.25. The fat content was determined according to ISO 1444/1996. The determination of ash content was realized according to AOAC (1995) method 920.153. The NaCl content was assessed by measuring chloride concentration with a Chloride Analyzer, Model 926, Sherwood Scientific Ltd., 1997.

### 2.3 Polycyclic aromatic hydrocarbons analyses

The 15 Polycyclic Aromatic Hydrocarbons (PAHs) were determined in the *kilichi* samples using a High‐Performance Liquid Chromatography coupled with a fluorescence detector (HPLC‐FLD) according to the method described by Brasseur et al., (2007). A Model 600 E solvent delivery system, equipped with a Model 717 automatic injector, a Mistral TM oven, and 2,475 Fluorescence detector (WATERS Corporation), was used. A C18 Pursuit 3 PAH (100 × 4.6 mm, 3 µm) equipped with a ChromGuard (10 × 3 mm) precolumn, both for Varian (Agilent Technologies) were used to separate the PAHs. The PAHs were extracted from the *kilichi* samples using the method described by Veyrand et al., (2007). The *kilichi* samples were frozen in liquid nitrogen and then freeze‐dried for 36–48H. For the extraction, one g of lyophilized *kilichi*, was homogenized with a mixture of hexane/acetone (50/50, v/v) by using the Accelerated Solvent Extraction system (ASE 200; Dionex). Then the solvent was evaporated until 1 ml, and then, reconstituted with 5 ml of cyclohexane. The reconstituted extract was then purified on a Chromatographic Column (Envi Chrom P Supelco), previously conditioned with 15 ml of ethyl acetate and 10 ml of cyclohexane. The extract was poured on the Column which was then washed two times with 3 ml of cyclohexane/ethanol (70/30, v/v) mixture. The PAHs were eluted three times using 4 ml of cyclohexane/ethyl acetate (40/60 v/v) mixture. The solvent was evaporated to dryness, then 90 µl of acetonitrile and 10 µl of the deuterated DiP‐D14, used as internal standard (LGC Promochem, France), were added. Twenty‐five (25) µl of this final extract was injected in the HPLC column. The limit of quantification (LOQ) corresponded to the first point of the calibration curve for each PAH and were 0.96 µg/kg of fresh weight for the Benzo (j) fluoranthene and Indeno [1, 2, 3‐cd] pyrene and 0.24 µg/kg fresh weight for the rest of the PAHs.

### 2.4 Microbiological analyses

Microbiological analyses were performed to determine the spoilage and pathogen germs like Aerobic Mesophilic Bacteria (AMB), Enterobacteriaceae, *Escherichia coli*, *Clostridium perfringens*, yeast and fungi, *Staphylococcus aureus*, *Bacillus cereus,* and *salmonella* spp*.* The analyses were performed on the primary dilution and its serial decimal dilutions according to ISO 6887‐1/1999 by using the peptone buffer water solution as dilution liquid. The enumeration of Aerobic Mesophilic Bacteria, Enterobacteria, and *E. coli* was realized according to ISO 4833/2003, ISO 21528‐2/2004, and ISO 16649‐2/2001, respectively. *Clostridium perfringens* was enumerated according to ISO 7937/2004 while *yeast* and *fungi* load was enumerated using ISO 21527‐2/2008. The enumerations of *Staphylococcus aureus* and *Bacillus cereus* were performed according to ISO 6888‐1/2003 and ISO 7932/2004, respectively. *Salmonella* were investigated using ISO 6579/2002.

### 2.5 Statistical analyses

The software SAS version 8 was used for statistical analysis of data. The means, medians, and standard deviations were calculated. The means comparisons were done with the test of Student at a threshold of 5% signification. The General Linear Model (GLM) was used for one‐way analysis of variance (ANOVA). The Least Square Mean was used to compare the two types of *kilichi*.

## 3 RESULTS AND DISCUSSION

### 3.1 Physicochemical characteristics of the *kilichi*

The results obtained from physicochemical analysis of both types of *kilichi* samples are presented in Table 2. The means of moisture content of *kilichi* are close to that obtained by Kalilou (1997) and Yacouba (2009), on both types of *kilichi* produced in Niger; and on coated *kilichi* produced in Nigeria and Cameroun (Apata, Osidibo, Apata, & Okubanjo, 2013; Jones, Tanya, Mbofung, Fonkem, & Silverside, 2001; Mgbemere et al., 2011; Olusola, Okubanjo, & Omojola, 2012). However, these values are lower than those obtained on Nigerian uncoated *kilichi* (19%–26% moisture content) (Raji, 2006)) and on dried *Bitong* (21.5%‐25.3%) (Petit, Caro, Petit, Santchurn, & Collignan, 2014). They are also in agreement with the standard (at least 88% of dry matter) stipulated by the National Council of Normalization of Niger (Conseil National de Normalisation du Niger—CNNN, 2004). According to Prescott, Harley, and Klein (2002), the microbial growth would be impossible in food products with water activity values lower than 0.7. So, the mean water activity values registered in the studied samples are low enough to avoid the growth of pathogenic bacteria in both types of *kilichi*. These results are in agreement with those obtained by Jones et al., (2001) for Cameroun coated *kilichi* (0.59) but lower than those of Petit et al. (2014) on dried *Bitong* (0.65–0.68), and Ratsimba et al., (2017) on dried *Kitoza* (0.86) and smoked *Kitoza* (0.94). *Bitong* is a traditional South African ready‐to‐eat meat product made from raw meat by salting, curing, and drying. There are two types, moist beef *Bitong* with high moisture content (higher than 40%) and dried *Bitong*. Kitoza is a traditional meat product from Madagascar, manufactured with strips of pork or beef. The process includes salting and mixing with spices followed by sun drying or smoking. Uncoated *kilichi* presents a mean pH lower than that of coated *kilichi.* The values found are similar to the results reported by Kalilou (1997), with a pH ranging between 5.7 and 6.2 for both types of *kilichi,* by Jones et al., (2001) for coated *kilichi* (5.81), by Petit et al., (2014) for dried *Bitong* (5.5–6.26) and by Ratsimba et al., (2017) for dry *Kitoza* and smoked *Kitoza* (5.67 and 5.87, respectively). These mean pH levels are lower than average value of 6.33 obtained by Eke et al. (2013) on *Dambu namma* (fried ground beef). The pH is an important parameter to control the sensory quality of meat. The average salt content in coated *kilichi* is higher than that in uncoated *kilichi*; this is justified by the addition of salt and bouillon cube in the seasoning sauce used to coat *kilichi*. The salt should play a role of preservative at short and average term but could be a source of disease. Petit et al., (2014) and Ratsimba et al., (2017) have reported salt contents of 5.5%–7.9%, 2.83%, and 3.61% in dried *Bitong*, dried *Kitoza,* and smoked *Kitoza,* respectively. The average protein content for both *kilichi* types are compliant with CNNN standard (50 à 70%) and the average coated *kilichi* protein content is similar to that obtained (49.8%) by Mgbemere et al., (2011). However, these results are lower than those obtained by Kalilou (1997), which are 64% protein content for coated *kilichi* and 74% for uncoated *kilichi*, and lower than those obtained by Jones et al., (2001), Olusola et al., (2012), and Apata et al., (2013), which are 61.95%, 62.33%, and 66.83% to 72.77% for coated *kilichi,* respectively. Also, the results showed that 06/16 (37.5%) coated kilichi had their protein content lower than the minimum (50%) recommended by the CNNN.

**Table 2:** Physicochemical characteristics of uncoated and coated *kilichi*

Compound	Uncoated *kilichi* (*n* = 14)					Coated *kilichi* (*n* = 16)				
	Min	Max	Mean	Median	*SD*	Min	Max	Mean	Median	*SD*
pH	5.3	6.4	5.76	5.7	0.31	5.60	6.4	6.05	6.08	0.24
a_w_	0.401	0.486	0.445	0.445	0.026	0.427	0.593	0.488	0.479	0.049
NaCl (% DM)	0.53	3.61	1.43	1.2	0.84	1.37	3.39	2.51	2.56	0.56
Moisture (%)	7.31	11.01	8.76	8.47	1.12	7.24	11.7	9.83	10.13	1.30
Protein (%)	62.74	84.48	72.32	72.48	5.83	37.66	62.1	51.23	50.71	6.63
Fat (%)	5.71	22.64	13.42	13.2	4.8	11.17	24.73	17.53	18.74	3.43
Ash (%)	4.12	6.95	5.19	4.97	0.89	5.12	8.47	6.24	6.19	0.80

Abbreviations: a_w_, water activity; DM, dry matter basis; Max, maximum; Min, minimum; *n*, number of samples analyzed; *SD*, Standard deviation.

The two types of *kilichi* are richer in protein than *Dambu namma* (fried ground beef) which values vary from 37.14% to 45.88% (Eke et al., 2013). Average values of fat content are compliant with CNNN standards (10 to 25%). However, 03/14 samples (21.43%) of uncoated *kilichi* are not compliant with CNNN standards, with a fat content lower than 10%. On one hand, fat contents of coated *kilichi* are higher than value (6.90%) reported by Olusola et al., (2012) and slightly higher than those obtained by Kalilou (1997), Mgbemere et al., (2011) and Apata et al., (2013); on the other hand, they are lower than value (25.39%) obtained by Jones et al., (2001). The difference between the two types of *kilichi* can be explained by use of many seasoning stuffs for coated *kilichi*. The fat content in *kilichi* should be low and of good quality; otherwise *kilichi* shelf life is short. Average ash values of the two types of *kilichi* are similar to that obtained by Yacouba (2009) (4.07% and 6.96%, respectively) and to that obtained by Mgbemere et al. (2011) and Jones et al., (2001) on the coated *kilichi* (5.2% and 6.2%, respectively) but lower than 10.31% reported by Olusola et al., (2012). These values on the other hand are higher than 3.70% obtained by Apata et al., (2013). The higher ash content of coated *kilichi* may be due to spices added.

The comparison of the two types of *kilichi* show a significant effect (*p* < .05) on moisture and fat, highly significant effects (*p* < .01) on pH and water activity, and very highly significant effects (*p* < .001) on salt, proteins, and ash contents (Table 3). The high value of the Mean standard error, shows the lack of standardization of the process. For *Kilichi* type production diverse types and quality of meat, different materials and practices, seasonings like spices which depend on producer are used (Boubacar et al., 2019).

**Table 3:** Comparison of the physicochemical characteristics of the two types of *kilichi*

Compound	Uncoated *kilichi* (*n* = 14)		Coated *kilichi* (*n* = 16)		*p* value
	LS mean	*SE*	LS mean	*SE*	
pH	5.75	0.07	6.06	0.06	.002**
a_w_	0.446	0.010	0.487	0.010	.008**
NaCl (%DM)	1.40	0.20	2.53	0.18	.0003***
Moisture (%)	8.77	0.34	9.82	0.32	.034*
Protein (%)	72.43	1.49	51.13	1.39	<.0001***
Fat (%)	13.40	1.14	17.54	1.07	.015*
Ash (%)	5.17	0.18	6.25	0.17	.0002***

Abbreviations: LS Mean, Least Squares Means; *SE*, Mean standard error.

*** *p* < .001, **p < .01 and *p < .05: significance.

### 3.2 Contamination of *Kilichi* samples by PAHs

Upon 15 EU PAHs considered as cancerogenic and genotoxic, 12 were detected and quantified in the two types of *kilichi*: Benzo[*a*]Anthracène (BaA); Benzo[*a*]pyrene (BaP); Benzo[*b*]fluoranthene (BbF); benzo[*c*]fluorene (BcF); Benzo[*gh*i]perylene (BgP); Benzo[*j*]fluoranthene (BjF); Benzo[*k*]fluoranthene (BkF); Chrysene (CHR); Dibenzo [*a,h*]anthracene (DhA); Dibenzo [*a,*e]pyrene (DeP); 5‐methylchrysene (5MC); and Indeno[1,2,3‐*cd*]pyrene (IcP) (Table 4). Dibenzo[a,h]pyrene (DhP), Dibenzo[a,i]pyrene (DiP), and Dibenzo[a,l]pyrene (DIP) have not been detected in the *kilichi* samples. Lorenzo, Purriños, Fontán, and Franco (2010) reported similar average values in smoked sausages including Androlla (36.45 µg/kg) and Botillo (29.39 µg/kg). Stumpe‐Vīksna, Bartkevičs, Kukāre, and Morozovs (2008) on the other hand found higher average values in smoked meats (47.94 to 470.91 µg/kg). The lower average values of PAHs registered in *kilichi* samples collected from Niger compared to that of the literature is probably due to the fact that some samples are collected from localities of Maradi where grilling is not applied. The different types of *kilichi* are contaminated in decreasing order by BaA, CHR, BcF, BbF, BjF, BaP, BkF, BgP, IcP, DeP, DhA, and 5MC. The distribution of these contaminants in coated *kilichi* represents 22.44%, 18.73%, 20.39%, 8.83%, 7.01%, 6.86%, 5.30%, 4.42%, 3.22%, 1.40%, 0.91%,and 0.57% of the total sum of PAHs respectively, against 19.38%, 18.99%, 15.59%, 10.13%, 9.07%, 8.46%, 3.30%, 6.08%, 4.93%, 2.29%, 0.75%, and 0.93% for uncoated *kilichi*. This distribution is similar to the relative proportion of the 15 EU reported by EFSA (2008) on 1,375 grilled or smoked meat products of many countries of the European Union and Veyrand et al., (2013) on 725 food products consumed in France.

**Table 4:** PAHs levels (µg/kg) in investigated *kilichi* samples collected in Niger

PAHs	Uncoated *kilichi* (*n* = 14)					Coated *kilichi* (*n* = 16)				
	Min	Max	Mean	Median	*SD*	Min	Max	Mean	Median	*SD*
BbF	0.85	8.16	2.30	1.62	1.88	<LOQ	8.9	3.40	2.43	2.65
DhA	<LOQ	0.52	<LOQ	<LOQ	<LOQ	<LOQ	0.87	0.35	0.31	0.24
BgP	0.47	4.54	1.38	1.12	1.02	0.26	4.4	1.70	1.44	1.29
DeP	<LOQ	1.93	0.52	<LOQ	0.57	<LOQ	1.41	0.54	0.53	0.44
BjF	<LOQ	6.56	2.06	1.39	1.52	<LOQ	6.52	2.70	2.2	1.95
BcF	1.16	10.73	3.54	2.65	2.55	<LOQ	18.84	7.85	5.98	6.05
BaA	1.66	10.64	4.40	3.91	2.36	1.98	23.13	8.64	6.62	5.92
CHR	1.65	9.16	4.31	3.95	2.22	1.59	25.86	7.21	6.16	5.67
5MC	<LOQ	1.48	<LOQ	<LOQ	<LOQ	<LOQ	0.94	<LOQ	<LOQ	<LOQ
BkF	0.31	1.81	0.75	0.55	0.44	<LOQ	10.03	2.04	0.94	2.63
BaP	0.67	6.59	1.92	1.3	1.63	0,32	7.06	2.64	2.27	1.94
IcP	<LOQ	2.41	1.12	<LOQ	<LOQ	<LOQ	3.1	1.24	1.11	0.86
Sum PAHs	9.74	64.13	22.67	18.42	14.74	6.88	97.28	38.54	34.54	24.39
PAH4	4.98	34.55	12.93	11.2	7.9	4.37	60.67	21.89	17.45	15.03

Abbreviations: LOQ, limit of quantification; Max, maximum; Min, minimum; PAH4, BbF + BaA+BaP + Chrysene; *SD*, Standard deviation.

The adsorption of PAHs depends on several factors, in particular the wood species used to generate the smoke (Lorenzo et al., 2010). Boubacar et al., (2019) reported 20 types of wood used by processors in grilling *kilichi* in Niger. This difference of PAHs can also be due to drying/smoking procedures and climate conditions (temperature and humidity) in the regions (Roseiro et al., 2012).

There was a significant (*p* < .05) difference between both types of *kilichi* as far as DhA, BcF, and BaA concentrations were concerned. Also, significant (*p* < .1) differences of BkF and the sum of PAHs were observed between both types of *kilichi* (Table 5). Benzo[*a*]Anthracène, Benzo[*c*]fluorene, and Chrysene are the most abundant contaminants regardless the type of *Kilichi*. However, these contaminants concentrations are higher in coated *kilichi* (1.5 to 2 times) than in uncoated *kilichi*. Coating is generally done by covering meat surface with peanut paste‐based sauce before grilling. The application of this sauce generally rich in fat on the meat before grilling could affect the level of contamination in PAHs which absorption is proportional to product fat content (Duedahl‐Olesen et al., 2015; Farhadian et al., 2010; Santos et al., 2011). In fact, while grilling, the fat melts and falls on the combustible thereby causing their pyrolysis and leading to the formation of PAHs.

**Table 5:** Comparison of PAHs profiles (µg/kg) in the two types of *kilichi* investigated in Niger

PAHs	Uncoated *kilichi*		Coated *kilichi*		*p* value
	LS mean	*SE*	LS mean	*SE*	
BbF	2.40	0.66	3.31	0.62	.330
DhA	0.18	0.05	0.34	0.05	.034*
BgP	1.43	0.33	1.66	0.31	.615
DeP	0.54	0.14	0.52	0.13	.906
BjF	2.15	0.49	2.63	0.46	.480
BcF	3.78	1.32	7.64	1.24	.044*
BaA	4.72	1.20	8.36	1.13	.038*
CHR	4.42	1.28	7.12	1.20	.139
5MC	0.20	0.10	0.23	0.10	.872
BkF	0.74	0.51	2.05	0.48	.074
BaP	1.99	0.51	2.57	0.48	.419
IcP	1.15	0.21	1.22	0.20	.803
Sum PAHs	23.70	5.72	37.64	5.35	.089
4 PAHs	13.54	3.43	21.35	3.20	.110

Abbreviations: LSMean, Least Squares Mean; *SE*, Mean standard error.

* *p* < 0, 05: significance.

The European Commission (Rule n°835/2011) fixed maximal content of 2 μg/kg for Benzo(a)pyrene (BaP) and 12 μg/kg for PAH4 (sum of (BaP; BaA; BbF and Chrysene) in food commodities like meat and meat products (EFSA, 2008). BaP concentrations contribute at 8.46% and 6.86% to the formation of the total sum of PAHs in uncoated and coated *kilichi,* respectively. BaP concentrations registered on *kilichi* are similar to those obtained by Manda et al., (2012) on fried meat (2.32 μg/kg) and Husam et al., (2011) on *Tikka* meat (2.48). Smaller values (0.14 to 1.10 μg/kg) have been reported by EFSA (2008), Roseiro et al., (2012), Santos et al., (2011), Farhadian et al., (2010); Rozentāle et al., (2015) and Matorell et al., (2010) on 2,145 meat products of EU countries including smoked meat products of Spain; *kebab*: smoked meat of Latvia and meat products of Catalonia (Spain). However, previous studies have recorded higher values (7 to 91 μg/kg) on smoked and grilled meat (*Satay*), Hamburgers meat, smoked *Gentile di maile* in Italy, *Suya,* and smoked *Shrimp* (Manda et al., 2012; Farhadian et al., 2010; Rose et al., 2015; Carrabs et al., 2014; Akpambang et al., 2009 and Kpoclou et al., 2013). The average concentration of PAH4 represents 57% of the total sum of PAHs for both types of *kilichi.*

Table 6 represents PAHs profile in analyzed *kilichi* samples with a toxicity threshold fixed at 2 µg/ kg for BaP and at 12 µg/ kg for PAH4. 56.3% of coated *kilichi* samples pass the toxicity threshold for BaP and 75% exceed the limit for PAH4. Also 28.6% of uncoated *kilichi* samples are not in accordance with the standard for BaP as well as for PAH4. Therefore, a consumer has 75% chance to consume a coated *kilichi* contaminated by the PAH4 and 56% by BaP against 29% for uncoated *kilichi*. Considering the Bench Mark Limit dose (BMDL_10_), an adult of 60 kg is exposed to an intoxication risk by BaP if he consumes more than 210 g of uncoated *kilichi* per day, and for the PAH4, maximal ingestion level is 156 g. For coated *kilichi*, the daily maximal ingestion for an adult of 60 kg is 162 g and 90g for BaP and PAH4, respectively.

**Table 6:** Occurrence of *kilichi* types meeting the BaP and PAH4 standards in the market

*kilichi* types	BaP (concentration)		Sum PAH4 (concentration)	
	≤2 µg/kg*	>2 µg/kg	≤12µg/kg**	>12 µg/kg
Uncoated *kilichi* (*n* = 14)	71.43%	28.57%	71.43%	28.57%
Coated *kilichi* (*n* = 16)	43.75%	56.25%	25.00%	75.00%

* Maximum limit of BaP set by European Union Commission Regulation N. 1881/2006.

** Maximum limit of sum 4PAHs set by European Union Commission Regulation N. 1881/2006.

### 3.3 Microbiological contamination of *kilichi*

The microbial counts in each type of *kilichi* and their compliance with CNNN (2004) and Public Health Laboratories (PHLS) (Gilbert et al., 2000) standards are given in tables 7 and 8. A total of 10/14 (71%) of uncoated *kilichi* samples are not compliant with standards against 100% of coated *kilichi*. For each type of *kilichi*, microbial load could come from a post‐treatment contamination because of application of high temperature of grilling for most *kilichi* samples and their low postprocessing water activity. The AMB load is an important indicator of general hygiene. Boubacar et al., (2019) reported that processors used inappropriate packaging for storage and sale of *kilichi*. The results also showed that l/16 (6%) of coated *kilichi* samples are not compliant with standards for *S. aureus*, *B. cereus*, *C. perfringens*, and 5/16 (31%) and 8/16 (50%) are not compliant for yeast and fungi, respectively. *E. Coli* and enterobacteria were not detected; however, *salmonella* was detected in one sample. A consumer has a 6% chance to be exposed to an intoxication by pathogens bacteria by consuming coated *kilichi*. For uncoated *kilichi*, the noncompliant samples for *S. aureus, C. perfringens,* yeasts*,* and fungi are in the proportions 2/14 (14%), 1/14 (7%), 3/14 (21%), and 12/14 (86%), respectively. Neither, *E. coli*, nor *B. Cereus* and enterobacteria were detected in any uncoated *kilichi* samples. These mean values obtained are lower than those reported by Mbawala et al., (2010) for spicy coated *kilichi* and not spicy coated *kilichi*. Raji, (2006) also reported higher mean values for uncoated *kilichi*. However, Jones et al., (2001) reported a lower value of AMB for coated *kilichi*. Averages of AMB, *E. coli, S. aureus* load counted on the two types of *kilichi* are lower than that counted on *Balangu* (grilled meat) (Moshood, Tengku, & Ibrahim, 2012) and on *Dambun nama* (fried ground beef) (Salihu et al., 2010).

**Table 7:** Microbial load (Log_10_ CFU/g) of uncoated *kilichi* samples and compliance with standards (*n* = 14)

Germs	Positive samples	Min	Max	Median	Mean ± *SD*	Acceptable limit	Noncompliant samples
AMB	14 (100%)	3.5	6.2	5	4.8 ± 0.8	4^a^	10 (71%)
*S. aureus*	2 (14%)	3.7	4.2	3.9	3.9 ± 0.38	2^b^	2 (14%)
*E. coli*	0 (0%)	<1	<1	<1	<1	2^b^	0 (0%)
*Enterobacteriaceae*	1 (7%)	<1	1.8	<1	<1	4^b^	0 (0%)
Molds	12 (86%)	<1	4.6	3	3.1 ± 1.2	2^a^	12 (86%)
Yeast	3 (21%)	<1	4	<1	1,3 ± 1,2	2^a^	3 (21%)
*B. cereus*	0 (0%)	<1	< 1	<1	<1	4^b^	0 (0%)
*C. perfringens*	5 (36%)	1.7	2.4	1.9	1.9 ± 0.3	2^b^	1 (7%)
*Salmonella spp*	Abs	Abs	Abs	Abs	Abs	Abs/25g^a^	0 (0%)

Abbreviations: Abs, Absence in 25 g; AMB, Aerobic Mesophilic Bacteria; Maxi, Maximum; Mini, Minimum; *N*, number of samples analyzed; Positive samples, Samples in which colonies are detected; *SD*, Standard deviation.

a According to CNNN standard.

b According to HPLS, 2000.

**Table 8:** Microbial load (Log_10_ CFU/g) of coated *kilichi* samples and compliance with standards (*n* = 16)

Germs	Positive samples	Min	Max	Median	Mean ± *SD*	Acceptable limit	Noncompliant samples
AMB	16 (100%)	4.6	6.8	5.2	5.3 ± 0.6	4^a^	16 (100%)
*S. aureus*	1 (6%)	<1	3.6	<1	<1	2^b^	1(6%)
*E. coli*	1 (6%)	<1	1	<1	<1	2^b^	0 (0%)
*Enterobacteriaceae*	4 (25%)	1	2.2	2	1.8 ± 0.6	4^b^	0 (0%)
Molds	10 (63%)	<1	4.6	2.5	2.4 ± 1.6	2^a^	8 (50%)
Yeast	5 (31%)	<1	3.7	<1	1.5 ± 1.2	2^a^	5 (31%)
*B. cereus*	1 (6%)	<1	3.4	<1	<1	4^b^	1(6%)
*C. perfringens*	5 (31%)	<1	2.2	<1	1.1 ± 0.6	2^b^	1(6%)
*Salmonella spp*	1 (6%)	Abs	1	–	–	Abs/25g^a^	1(6%)

Abbreviations: Abs, Absence in 25 g; AMB, Aerobic Mesophilic Bacteria; Maxi, Maximum; Mini, Minimum; *N*, number of samples analyzed; Positive samples, Samples in which colonies are detected; *SD*, Standard deviation.

a According to CNNN standard.

b According to HPLS, 2000.

For all germs investigated, coated *kilichi* present higher loads than uncoated *kilichi*. This difference could be due to germs carried by seasoning spices. Shamsuddeen (2009), Frazier and Westhoff (2006), have reported that some spices do not have antimicrobial activity, so that the meat treated with spices could have high microbial load. In the same logic, Price and Schweigert (1971) stated that unless the spices are treated to reduce their microbial load, they can be a source of high number of undesirable germs in the product to which they are added. The presence of *S. aureus, Enterobacteria, C. perfringens, E. coli,* and *Salmonella* in the products is an indication of poor hygienic practices during processing. Edema, Osho, and Diala (2008) detected in spices used in the preparation of Suya (grilled meat product) the following microbial flora: *AMB* 3.42–3.50 × 10^5^ cfu/g, yeast *and fungi* 1.27–1.43 × 10^5^ cfu/g, *Coliforms* 0.37–0.47 × 10^5^ cfu/g, *Staphylococcus* 0.37 × 10^5^ cfu/g, *Bacillus cereus* 0.02 × 10^5^ cfu/g, *Salmonellas* 0.37–0.43 × 10^5^ cfu/g. These results confirm the high AMB load obtained in coated kilichi.

## 4 CONCLUSION

This study on the physicochemical characteristics and safety of *kilichi* produced in five regions of Niger reveals that the microbiological quality of the studied samples is generally not satisfactory. The pathogen bacteria responsible of the food intoxication (*B. cereus, S. aureus, and Salmonella* spp) and germs indicators (100% coated *kilichi* and 70% uncoated *kilichi*) of nonrespect of hygiene practices were detected despite the unfavorable conditions (a_w_ < 0.7) for their growth. Coated *kilichi* has a level of contamination higher than uncoated *kilichi*. As far as chemical contamination is concerned, to our knowledge, this preliminary study reports for the first time the level of aromatic polycyclic hydrocarbons contamination of *kilichi* in Niger. High levels of BaP (7.06 µg/kg) and PAH4 (60.67 µg/kg) contamination recorded in coated *kilichi*, indicators of PAHs toxicity, showed potential health risks for consumers. Nutritionally, uncoated *kilichi* is richer in proteins than coated *kilichi*. However, coated *kilichi* is richer in lipids and in salt which high level can cause public health problems. The study showed that the manufacturing of the product is not standardized for each type of *kilichi*. Based on these results, future studies should be conducted for the implementation of an improved and standardized process of traditional *kilichi*. This will enable to obtain a product of better quality and safety.

## CONFLICT OF INTEREST

The authors declare that they have no conflict of interest.

## ETHICAL APPROVAL

This study does not involve any human or animal testing.
